# Patient characteristics and outcomes of left ventricular assist device implantation on early versus late weekdays

**DOI:** 10.1016/j.xjon.2023.11.001

**Published:** 2023-11-11

**Authors:** Amit Alam, Johanna S. van Zyl, Melissa Medina, Katharina Fetten, Aldo E. Rafael, Joost Felius, Dan M. Meyer, Shelley A. Hall

**Affiliations:** a Department of Cardiology, New York University, New York, NY; b Texas A&M University Health Science Center College of Medicine, Dallas, Tex; c Baylor Scott & White Research Institute, Dallas, Tex; d Center for Advanced Heart and Lung Disease, Baylor University Medical Center, Baylor Scott and White Health, Dallas, Tex

**Figure:**
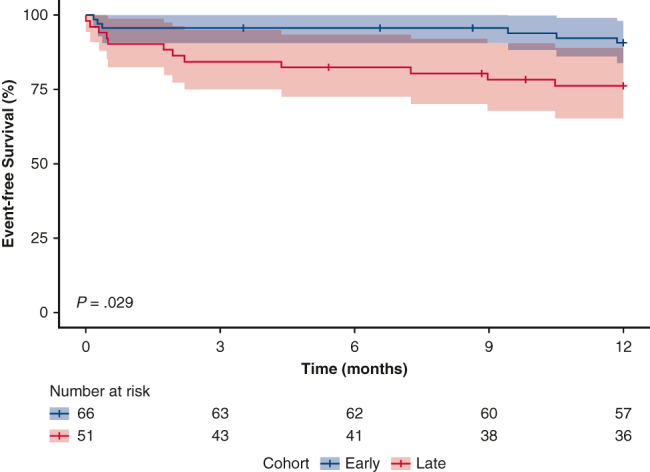
There is lower event-free survival for patients implanted with an LVAD late in the week.

**Central Message**

Patients implanted with an LVAD late in the week may be more likely to have renal adverse events, longer lengths of stay, and lower event-free survival than those implanted earlier in the week.

Previous studies reported worse outcomes when procedures occur during “off hours” (holidays, weekends, and nights).^1^ This “off-hours effect” is likely more significant in the treatment of acute, deadly diseases such as cardiogenic shock (CS), when small deviations in timing could have a large impact on patient morbidity and mortality. Left-ventricular assist device (LVAD) implantations are usually scheduled as weekday procedures, but little is known about differences in preoperative status and outcomes in those implanted on early compared with late weekdays.

## Methods

In a retrospective review of LVAD recipients in our prospectively maintained single-institutional Interagency Registry for Mechanically Assisted Circulatory Support (Intermacs) database, we compared differences in patient characteristics and outcomes between those implanted during early (Monday-Wednesday) and late (Thursday-Friday) weekdays. Patients implanted between January 2017 and March 2022 were included. This study was approved by the institutional review board of Baylor Scott & White Research Institute under an umbrella protocol for retrospective research with consent form waiver (IRB File No. 011-274, November 30, 2022).

The primary outcome was a composite of survival to recovery or transplant free of debilitating stroke, similar to landmark LVAD trials (eg, Mehra and colleagues^2^), debilitating stroke with modified Rankin scale >3 included for moderately severe-to-severe disability. Length of stay (LOS) post-LVAD was calculated from implant to discharge dates. Complications up to 1-year postimplant were collected from adverse events defined according to Society of Thoracic Surgeons Intermacs, version 6.1, definitions.^2^

Patient characteristics, described using proportions or median and quartiles, were compared between early to late weekday cohorts using Wilcoxon rank sum, χ^2^, or Fisher exact tests. Time to mortality or debilitating stroke was censored if the LVAD was explanted for recovery, exchange or transplant, and compared up to 1-year postimplant using Kaplan–Meier analysis with log-rank tests. The hazard ratio (HR) was estimated with Cox proportional hazards models. Analyses were performed in R (Version 4.2; R Foundation for Statistical Computing) and significance assessed 2-sided at a threshold <.05.

## Results

Of 117 patients included, 66 and 51 patients were implanted on early and late weekdays, respectively. Patient characteristics were comparable (Table 1). Four additional patients implanted on Saturday were excluded.

**Table 1:** Patient characteristics and outcomes comparing LVAD implants on early (Monday-Wednesday) versus late weekdays (Thursday-Friday)

Variable	Overall (n = 117)	Early (n = 66)	Late (n = 51)	*P* value
Age, y	59 [50-68]	59 [51-66]	59 [51-69]	.53
Sex, male	96 (82%)	56 (85%)	40 (78%)	.51
Race				
African American	37 (32%)	18 (27%)	19 (37%)	.34
White	78 (67%)	46 (70%)	32 (63%)	.55
BMI, kg/m^2^	28 [24-32]	28 [24-32]	27 [25-31]	.69
Bridge to transplant	22 (19%)	13 (20%)	9 (18%)	.97
Severe diabetes	3 (3%)	2 (3%)	1 (2%)	1.00
Chronic renal disease	25 (21%)	12 (18%)	13 (25%)	.47
Ischemic cardiomyopathy	65 (58%)	37 (60%)	28 (56%)	1.00
Intermacs profile				.59
1	19 (16%)	12 (18%)	7 (14%)	
2	50 (43%)	29 (43%)	21 (41%)	
3	40 (34%)	19 (29%)	21 (41%)	
4+	8 (7%)	6 (9%)	2 (4%)	
Reason for admission, planned VAD placement	41 (35%)	25 (38%)	16 (31%)	.59
Temporary mechanical support	32 (27%)	18 (27%)	17 (33%)	.36
Device brand				.66
HeartMate 3	89 (76%)	51 (77%)	38 (75%)	
HeartMate II	11 (9%)	7 (11%)	4 (8%)	
Medtronic HVAD	17 (15%)	8 (12%)	9 (18%)	
In-hospital outcomes				
LOS post-LVAD, d	15 [11-20]	14 [11-17]	18 [12-23]	**.04**
In-hospital mortality	7 (6%)	3 (5%)	4 (8%)	.70
Major bleeding	5 (4%)	2 (3%)	3 (6%)	.65
Major infection	6 (5%)	5 (8%)	1 (2%)	.23
Device-related infection	0 (0%)	0 (0%)	0 (0%)	1.00
Renal dysfunction	3 (3%)	1 (2%)	2 (4%)	.58
Right heart failure	9 (8%)	3 (5%)	6 (12%)	.17
Stroke	5 (4%)	3 (5%)	2 (4%)	1.00
Debilitating stroke (mRS >3)	2 (2%)	1 (2%)	1 (2%)	1.00
1-y outcomes				
Death	17 (15%)	6 (9%)	11 (22%)	.10
Readmitted	64 (55%)	35 (53%)	29 (57%)	.82
Number of readmissions per patient	1 [0-1]	1 [0-1]	1 [0-3]	.21
Total number of days readmitted	10.5 [2-26.2]	7 [1.5-22.5]	17 [3-37]	.09
Major bleeding	23 (20%)	11 (17%)	12 (24%)	.49
Major infection	33 (28%)	18 (27%)	15 (29%)	.96
Device-related infection	4 (3%)	2 (3%)	2 (4%)	1.00
Renal dysfunction	11 (9%)	1 (2%)	10 (20%)	**.001**
Right heart failure	19 (16%)	8 (12%)	11 (22%)	.26
Stroke	11 (9%)	4 (6%)	7 (14%)	.21
Debilitating stroke (mRS >3)	4 (3%)	1 (2%)	3 (6%)	.32
Death or debilitating stroke	18 (15%)	6 (9%)	12 (24%)	.04

Categorical data is summarized as the frequency (%) and continuous data as the median [quartile 1-quartile 3]. *P* values less than .05 is significant indicated in bold. *BMI*, Body mass index; *Intermacs*, Interagency Registry for Mechanically Assisted Circulatory Support; *VAD*, ventricular assist device; *HVAD*, HeartWare Ventricular Assist Device; *LOS*, length of stay; *mRS*, modified Rankin scale; *LVAD*, left ventricular assist device.

Table 1 shows outcomes and complications during the index hospitalization. Patients in the late week cohort had longer post-LVAD LOS (median 18 vs 14 days, *P* = .04). Other complications analyzed including in-hospital mortality, major bleeding, major infection, right heart failure, renal dysfunction, and stroke did not differ significantly.

**Figure 1:**
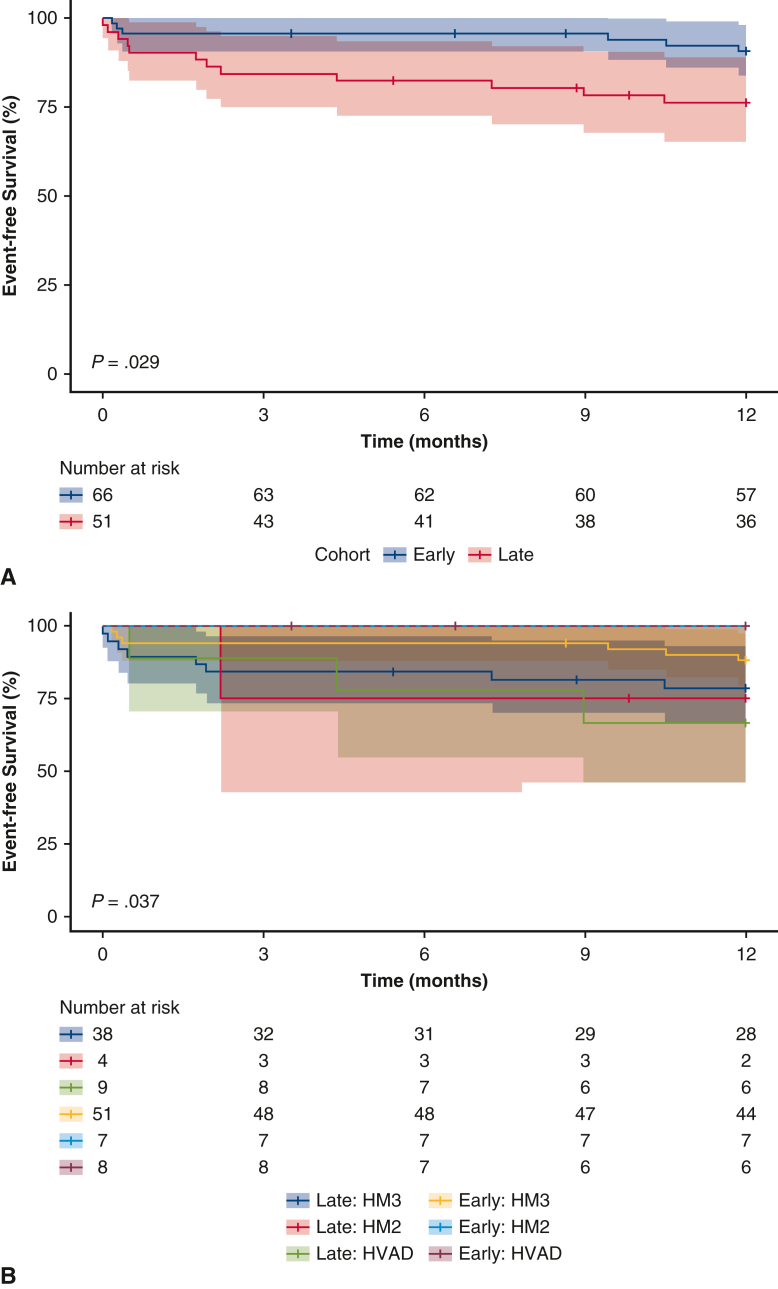
Comparison the Kaplan–Meier curves with 95% confidence bands of the primary composite outcome of event-free survival to recovery or transplant up to 1-year postimplant between early-versus late-week LVAD implants (A) overall and (B) stratified by device type. The primary outcome is a composite of mortality or debilitating stroke (modified Rankin score >3). *LVAD*, Left ventricular assist device.

At 1-year postimplantation, more patients in the late weekday cohort experienced renal dysfunction (20% vs 2%, *P* = .001), whereas other complications did not differ significantly. (Table 1). Eighteen patients died and/or experienced a debilitating stroke, of whom 6 (9%) and 12 (24%) were in the early and late weekday cohorts, respectively. Survival free from debilitating stroke was lower in the late weekday relative to early weekday cohorts (*P* = .029, stratified by device type *P* = .037; Figure 1) with a 2.85 increase in the risk of the composite outcome (HR; 95% confidence interval [CI], 1.07-7.59). The majority (11/18) of the composite outcome events occurred within the first 3 months post-VAD. Complications occurring within the first 3 months that were associated with the composite outcome were major infections (HR, 4.5; 95% CI, 1.7-11.6, *P* = .002) and renal dysfunction (HR, 4.1; 95% CI, 1.2-14.3, *P* = .03).

## Discussion

Our study is the first to report differences in outcomes in late-week LVAD implants. Patients implanted in on late weekdays had (1) longer LOS post-LVAD, (2) more renal adverse events at 1 year, and (3) lower rates of survival or debilitating stroke than those implanted earlier in the week.

For those implanted later in the week, postoperative care within the first 48 hours of implant may be impacted by a “weekend” effect. Optimal level of care may be harder to achieve on the weekend due to lack of staffing and resource limitations during off hours with “on-call” and “more-experienced” staff available only for emergency situations.^3^ Thus, our late weekday patients’ experience and outcomes may show similarities with those admitted over the weekend.

A weekend effect on renal outcomes was also reported previously in patients with CS.^4^ Renal dysfunction occurred early on at 3 months and persisted at 1 year following implantation. Worsening peri- and postoperative renal function is perhaps a surrogate of sicker patients not detected by our study and warrants further exploration.

Currently, one of the leading methods to improve outcomes associated with CS is improvement of treatment protocols.^5^ At our center (Baylor University Medical Center, Dallas, Tex), we have recently added 24/7 in-house heart failure attending coverage (in addition to 24/7 in house critical care attending coverage), daily multidisciplinary team rounding, and having system-wide protocols to guide timely transfers into the cardiac intensive care unit when warranted. In the setting of LVAD placement, the availability of expert care may eliminate deficits in patient care, allow for definitive planning regardless of implantation time and further provide immediate intervention for acute complications. Although we realize that dual in-house 24/7 attending coverage is not possible in all institutions, we propose that each institution periodically review their outcomes and modify protocols based on resources available to deliver optimal patient care regardless of the day of the week.^5^

There are several limitations to the study, including a retrospective single-center investigation with a modest sample size, so our results may not be generalizable to other centers. The sample size was sufficient to detect a difference in the primary outcome but may have limited power to detect smaller rate differences (eg, complication rate differences smaller than 25% when one group has a rate of 50%) between groups. In addition, some factors including team experience, on-call schedule, and staff fatigue may influence patient outcomes but are not captured in our medical record and thus were not included in this analysis.

## Conclusions

Patients implanted with LVADs late in the week may be more likely to have renal adverse events at 1 year and longer length of stay following LVAD implantation with lower event-free survival than those who received an LVAD earlier in the week. Analysis of the full Intermacs database to ascertain if these results are generalizable is warranted.

## Conflict of Interest Statement

A.A. reported speaker for Abbott and CareDx. S.A.H. reported consultant/advisor for Abbott, CareDx, Natera, Evaheart, and Abiomed. All other authors reported no conflicts of interest.

The *Journal* policy requires editors and reviewers to disclose conflicts of interest and to decline handling or reviewing manuscripts for which they may have a conflict of interest. The editors and reviewers of this article have no conflicts of interest.
